# Lack of awareness of Hepatitis B screening and vaccination in high-risk groups

**DOI:** 10.3906/sag-2008-18

**Published:** 2021-06-28

**Authors:** H. Yasemin BALABAN, Abdullah Tarık ASLAN, Şefika AYAR, Osman DAĞ, Alpaslan ALP, Cem ŞİMŞEK, Cavanşir VAHABOV, Tolga YILDIRIM, Hakan GÖKER, Yahya BÜYÜKAŞIK, Halis ŞİMŞEK

**Affiliations:** 1 Department of Gastroenterology, Faculty of Medicine, Hacettepe University, Ankara Turkey; 2 Department of Internal Medicine, Faculty of Medicine, Hacettepe University, Ankara Turkey; 3 Department of Biostatistics, Faculty of Medicine, Hacettepe University, Ankara Turkey; 4 Department of Microbiology and Clinical Microbiology, Faculty of Medicine, Hacettepe University, Ankara Turkey; 5 Department of Nephrology, Faculty of Medicine, Hacettepe University, Ankara Turkey; 6 Department of Hematology, Faculty of Medicine, Hacettepe University, Ankara Turkey

**Keywords:** Vaccination, Hepatitis B virus, immunity

## Abstract

**Background/aim:**

Hepatitis B virus (HBV) vaccination rates are insufficient in high-risk patients worldwide. This study aimed to investigate the screening, immunization, and vaccination rates in three high-risk groups for HBV infection: allogeneic hematopoietic stem cell transplantation (AHSCT), renal transplantation (RT), and chronic hepatitis C (CHC) groups.

**Materials and methods:**

The serological data of consecutive patients between 2014 and 2019 were reviewed using the hospital database.

**Results:**

The HBV screening rates were 100.0%, 90.4%, and 82.4% in the AHSCT, CHC, and RT groups, respectively (p *=* 0.003). The immunization rates against HBV through either previous exposure or vaccination were 79.5%, 71.7%, and 46.5% in the AHSCT, RT, and CHC groups, respectively (p < 0.001). The HBV vaccination rate was significantly low in the CHC group (71.5%, 69.0%, 34.6% in the AHSCT, RT, and CHC groups, respectively, p < 0.001). If patients lost their immunity due to immunosuppressive therapy were accounted, the vaccination rates increased to 95.2% in the AHSCT group and 72.9% in the RT group. The rate of annual screening for HBV status was 97.9% in the AHSCT group, but it was only 23.9% in the RT group.

**Conclusion:**

HBV screening and vaccination rates were significantly lower in the RT and CHC groups than in the AHSCT group.

## 1. Introduction

Hepatitis B virus (HBV) infections result in acute or chronic hepatitis. Acute HBV infection can lead to liver failure, and chronic HBV infection can progress to cirrhosis, liver failure, liver cancer, and death. Thus, HBV infection has a significant burden on the healthcare system due to its high morbidity and mortality rates and treatment costs. Considering that almost half of adult patients are asymptomatic and unaware of their HBV infections, HBV transmission and persistence within the population are still a major public health problem with a significant burden [1]. According to the World Health Organization (WHO) report, 240 million people test positive for hepatitis B surface antigen (HBsAg); thus, they are diagnosed with chronic HBV infection World Health Organization. Hepatitis B Vaccines: WHO Position Paper [online]. Website http://www.who.int/wer/en/ [accessed 7 July 2017].. The prevalence of HBV infection significantly varies worldwide. Turkey, a country with intermediate endemicity, has an HBV infection prevalence between 2% and 8% [2–5].

Vaccination is the most effective method of preventing vertical and horizontal transmission of HBV infection. Therefore, HBV vaccination is strictly recommended in some high-risk adults who frequently receive blood or blood products, patients on dialysis, patients with diabetes, recipients of solid organ transplants, patients with chronic liver disease and human immunodeficiency virus infection, individuals working in prisons, individuals injecting drugs, household and sexual contacts of individuals diagnosed with chronic HBV infection, men who have sex with men, individuals with multiple sexual partners, healthcare workers, and others who may be exposed to blood, blood products, or other potentially infectious body fluids during their work.

Patients with a history of renal transplantation (RT), allogeneic hematopoietic stem cell transplantation (AHSCT), or chronic hepatitis C (CHC) must be vaccinated against HBV without missing any single patient. Furthermore, the ACIP recommended annual HBV screening in AHSCT and RT recipients [6]. The data for global vaccination rates in the high-risk groups are insufficient, although some studies have reported unacceptably low vaccination rates even in the high-risk groups [7,8]. These disappointingly low vaccination rates in the high-risk groups emphasized that we should review our practices for HBV screening and vaccination to achieve the WHO 2030 target of eliminating HBV worldwide. In this study, we aimed to investigate the current HBV vaccination and immunization status of the aforementioned high-risk patients.

## 2. Materials and methods

The HBV serology data of patients recruited to another study were used after a waiver of informed consent was obtained (Hacettepe University local ethics committee study approval identification code: GO 18/186). Patients aged ≥18 years with a history of RT (159 recipients), AHSCT (47 recipients), and CHC (83 patients) recruited from the nephrology, hematology, and gastroenterology departments, respectively, were included in the study. The data for HBV serology and HBV deoxyribonucleic acid (DNA) from January 1, 2014 to June 30, 2019 were reviewed to identify the HBV screening, vaccination, and immunization status of these patients. Patients who were not screened were excluded from the analysis of immunization and vaccination rates. The vaccination rate analysis also excluded patients with chronic HBV infection, occult hepatitis B, and HBV immunity gained by previous exposure. The vaccination status was obtained by face-to-face interviews or telephone calls in patients who were anti-HBs antibody positive but were not tested for total anti-HBV core antigen (HBc) antibody.

### 2.1. Definitions

**HBV screening:** Patients underwent HBsAg, anti-HBs, and anti-HBc total antibody testing.

**Screening rates were calculated as follows:**

Number of screened patients

Number of all patients in that group

**HBV immunization:** Patients had ≥10 mIU/mL anti-HBs antibody titers and either tested positive or negative for total anti-HBc antibody. In other words, HBV immunization suggested that anti-HBs positivity was gained through either vaccination or infection.

**Immunization rates were calculated as follows:**

Number of anti-HBs-positive patients

Number of all patients in that group - (chronic HBV infection + non-screened patients)

Hence, the following patients were excluded from the denominator value:

· Patients who were not screened at all.

· Patients who had chronic HBV infection.

**HBV vaccination:** Patients had ≥10 mIU/mL anti-HBs antibody titers and tested negative for total anti-HBc antibody.

**Vaccination rates were calculated as follows:**

Number of patients who are anti-HBs positive and anti-HBc total negative

Number of patients who have indication(s) for HBV vaccination

In the denominator, the following patients were excluded:

**·** Patients who were not screened at all.

**·** Patients who had chronic HBV infection.

**·** Patients who had occult hepatitis B.

**·** Patients who had immunity against HBV gained by previous exposure.

**Occult hepatitis B:** Patients tested positive for total anti-HBc antibody, together with negativity for HBsAg (anti-HBs and HBV DNA can be positive or negative).

**Chronic Hepatitis B infection:** Patients had positive HBsAg and anti-HBc total (HBV DNA can be positive or negative).

Serological markers of HBV infection were determined using commercially available immunoassays throughout the study. All assays were performed according to the manufacturers’ instructions. HBsAg, anti-HBs, anti-HBc immunoglobulin M, and anti-HBc immunoglobulin G were determined using chemiluminescent microparticle immunoassays via the Architect system (Abbott Laboratories, USA). HBV DNA was evaluated quantitatively by Cobas AmpliPrep real-time polymerase chain reaction (Roche Diagnostic Systems Inc., Germany). HBV DNA levels expressed as copy mL–1 were converted to IU mL–1 for standardization according to the WHO standards as directed by the manufacturer.

### 2.2. Statistical analysis

Frequency and percentages were reported for categorical variables. Fisher’s exact test was used for comparing the three independent groups in terms of categorical variables. Moreover, the same test was applied for post-hoc analysis by adjusting the p-values with Bonferroni correction. A two-sided p-value <0.05 was considered significant. All statistical analyses were performed using the Statistical Package for the Social Sciences (SPSS) version 23.0 (IBM Corp., Armonk, NY, USA) for Windows.

## 3. Results

### 3.1. Screening rates

The HBV screening rates were 100.0%, 90.4%, and 82.4% in the AHSCT, CHC, and RT groups, respectively (Figure). The screening rates were significantly different between the three groups (p = 0.001), with the AHSCT group achieving the highest screening rate among the three groups (p = 0.002). Additionally, the annual screening rate was 97.9% in the AHSCT group, but it was only 23.3% in the RT group.

**Figure:**
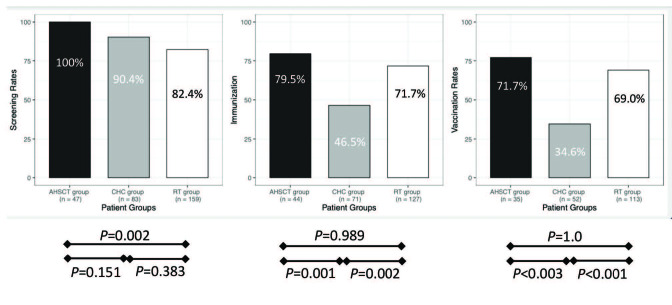
Hepatitis B virus screening, immunization, vaccination rates. The screening, immunization, and vaccination rates of AHSCT, RT, and CHC groups are shown in this figure. AHSCT: Allogeneic hematopoietic transplant recipients, RT: Renal transplant recipients, CHC: Chronic hepatitis C patients.

### 3.2. Immunization rates

The immunization rates were 79.5%, 71.7%, and 46.5% in the AHSCT, RT, and CHC groups, respectively (Figure). The immunization rates were significantly different among the three groups ( *P* < 0.001). The CHC group had significantly lower HBV immunization rate than the AHSCT and RT groups (Figure). Immunity was gained by previous exposure in 10.2%, 18.1%, and 21.1% of RT recipients, AHSCT recipients, and CHC patients, respectively.

### 3.3. Vaccination rates

The vaccination rates were far below the ideal rate of 100%. Specifically, the CHC group had significantly lower vaccination rate than the other two groups (Figure). Considering patients who were previously vaccinated but lost their immunity due to immunosuppressive drugs, the HBV vaccination rates increased to 95.2% and 72.9% in the AHSCT and RT groups, respectively. Immunity was lost during follow-up in 8 (18.1%) and 5 (3.9%) patients in the AHSCT and RT groups, respectively, all of whom gained anti-HBs positivity through vaccination. In contrast to RT patients who lost their immunity against HBV, all patients with AHSCT were revaccinated. This further emphasizes the importance of annual screening for HBV in the high-risk groups.

Occult hepatitis B and chronic HBV infections were detected in 1 (0.7%) and 4 (3.0%) patients undergoing RT, 1 (2.1%) and 3 (6.3%) patients undergoing AHSCT, and 4 (5.3%) and 4 (5.3%) patients with CHC, respectively. Compensated cirrhosis was observed in 19 (22.8%) patients with CHC. Significantly, only 1 of 6 (16.7%) patients with cirrhosis indicated for HBV vaccination was vaccinated.

## 4. Discussion

Although HBV vaccination is the most efficient method of eliminating HBV infection worldwide, this study revealed that the screening and vaccination rates were far from the ideal rates in the high-risk groups, and annual screening rate was significantly low among RT recipients. The high rate of acquisition of HBV immunity gained through previous exposure (10.2%, 18.1%, and 21.1% in the RT, AHSCT, and CHC groups, respectively) suggested that HBV infection is still an important health problem among the high-risk groups in Turkey.

Although the AHSCT group was managed according to the Centers for Disease Control and Prevention/ACIP guidelines [9], the RT and CHC groups had significantly low screening (82.4% and 90.4%, respectively) and vaccination rates (69.0% and 34.6%, respectively). The vaccination rate was 95.2% in the AHSCT group when patients who lost their immunity due to immunosuppressive treatment were considered. The high screening and vaccination rates in the AHSCT group can be possibly attributed to the protocol-based approach used in all AHSCT recipients in the pretransplant and posttransplant periods. Furthermore, the posttransplant annual HBV serology screening rate was 97.9% in the AHSCT group, which is possibly attributed to the dedicated nurses working in the hematology department. In the RT group, both the screening and vaccination rates were significantly lower than the recommended levels. Although long-term follow-up after transplantation is performed by a nephrologist in RT recipients, a significantly low (23.3%) annual screening rate of HBV serology is observed in these RT recipients. Hence, patients who lost their immunity during follow-up are possibly difficult to determine and thus, are not usually revaccinated. Similar to the RT group, the vaccination rates of CHC patients (46.5%) in the gastroenterology department and patients with cirrhosis (16.7%) were significantly low. These results emphasized the importance of dedicated healthcare personnel (mostly nurses) and the appropriate application of routine protocols to achieve high HBV vaccination rate in the high-risk groups. The availability of dedicated healthcare staff who continuously assess the HBV vaccination status of patients and who consistently remind doctors about HBV vaccination and the preparation of checklists and obligatory orders for HBV vaccination needs to be established in hospitals. The education programs directed to patients and healthcare staff might improve HBV screening and vaccination compliance. Launay et al. [10] conducted pre-post cluster randomized study to investigate the effects of training of healthcare staff, free on-site vaccine availability, and the combination of these two interventions. Interestingly, training of healthcare staff was not associated with any beneficial effect on neither vaccination acceptance nor coverage. However, the group receiving the combination of two interventions had significantly higher vaccination acceptance than the free on-site vaccination group. Therefore, training of healthcare staff and free on-site vaccine availability are important issues that need to be addressed to increase HBV vaccination coverage. Although vaccination is free of charge in Turkey, HBV vaccination rates were unacceptably low in the RT and CHC groups. Therefore, in real-life clinical practice, several challenges are encountered, which should be solved to achieve the recommended HBV vaccination rates.

Considering the significantly high HBV exposure risk, HBV vaccination is required in the specific high-risk groups. In a study comprising 1257 hepatitis C virus ribonucleic acid-positive subjects in the United States of America, the HBsAg positivity rate was 5.8%, and 58.8% of the patients were previously exposed to HBV [11]. The prevalence of HBV infection in RT patients was 4.2% according to a study from Brazil, and immunosuppression after RT led to HBV activation and liver-related complications [12]. Çakar et al. [13] showed that out of the 197 AHSCT recipients, 6.1% was HBsAg positive. Consistent with previous reports, our study demonstrated that HBV exposure was frequently observed in RT recipients (13.7%), AHSCT recipients (25.5%), and CHC patients (30.6%). Occult hepatitis B and chronic HBV infections were observed in 0.7% and 3.0% in the RT, 2.1% and 6.3% in the AHSCT, and 5.3% and 5.3% in the CHC groups, respectively. HBV immunity was gained by previous exposure in 10.2%, 18.1%, and 21.1% of patients in the RT, AHSCT, and CHC groups, respectively. Patients who gained their immunity by vaccination were at high risk of losing their immunity due to immunosuppressive treatment. Immunity was lost during follow-up in 18.1% of patients with AHSCT and 3.1% of patients with RT, emphasizing the importance of annual serological screening in these high-risk groups.

In conclusion, previous HBV exposure was substantially frequent in all high-risk groups. Although the screening rates for HBV were greater than 80%, the vaccination rates were far from the recommended rate, particularly for the RT and CHC groups. Furthermore, the loss of immunity gained by vaccination due to immunosuppressive treatment necessitates annual screening and revaccination. The lack of awareness for HBV infection in the high-risk groups requires further studies to identify the challenges encountered during HBV screening and vaccination to achieve the WHO 2030 target. Furthermore, multifaceted awareness campaigns should be organized to improve HBV vaccination rate in high-risk groups in Turkey. 

## Informed Consent

In this study, a waiver of informed consent was obtained from Hacettepe University Local Ethics Committee with a study approval identification code: GO 18/186.

## Funding

This research did not receive any specific grant from funding agencies in the public, commercial, or not-for-profit sectors.
